# Soil Fungal Resources in Annual Cropping Systems and Their Potential for Management

**DOI:** 10.1155/2014/531824

**Published:** 2014-08-28

**Authors:** Walid Ellouze, Ahmad Esmaeili Taheri, Luke D. Bainard, Chao Yang, Navid Bazghaleh, Adriana Navarro-Borrell, Keith Hanson, Chantal Hamel

**Affiliations:** ^1^Semiarid Prairie Agricultural Research Centre, Agriculture and Agri-Food Canada, P.O. Box 1030, 1 Airport Road, Swift Current, SK, Canada S9H 3X2; ^2^Department Food and Bioproduct Sciences, College of Agriculture and Bioresources, University of Saskatchewan, 51 Campus Drive, Saskatoon, SK, Canada S7N 5A8; ^3^Department of Soil Science, College of Agriculture and Bioresources, University of Saskatchewan, 51 Campus Drive, Saskatoon, SK, Canada S7N 5A8

## Abstract

Soil fungi are a critical component of agroecosystems and provide ecological services that impact the production of food and bioproducts. Effective management of fungal resources is essential to optimize the productivity and sustainability of agricultural ecosystems. In this review, we (i) highlight the functional groups of fungi that play key roles in agricultural ecosystems, (ii) examine the influence of agronomic practices on these fungi, and (iii) propose ways to improve the management and contribution of soil fungi to annual cropping systems. Many of these key soil fungal organisms (i.e., arbuscular mycorrhizal fungi and fungal root endophytes) interact directly with plants and are determinants of the efficiency of agroecosystems. In turn, plants largely control rhizosphere fungi through the production of carbon and energy rich compounds and of bioactive phytochemicals, making them a powerful tool for the management of soil fungal diversity in agriculture. The use of crop rotations and selection of optimal plant genotypes can be used to improve soil biodiversity and promote beneficial soil fungi. In addition, other agronomic practices (e.g., no-till, microbial inoculants, and biochemical amendments) can be used to enhance the effect of beneficial fungi and increase the health and productivity of cultivated soils.

## 1. Introduction

Microorganisms are involved in fundamental processes such as soil formation and nutrient cycling and can be seen as the cornerstone of the biosphere. They are an essential link between soil nutrient availability and plant productivity as they are directly involved in the cycling of nutrients through the transformation of organic and inorganic forms of nutrients. Certain microorganisms, in particular those interacting physically with plants in the rhizosphere, can also influence plant productivity negatively by causing disease or positively by enhancing plant growth.

**Figure 1:**
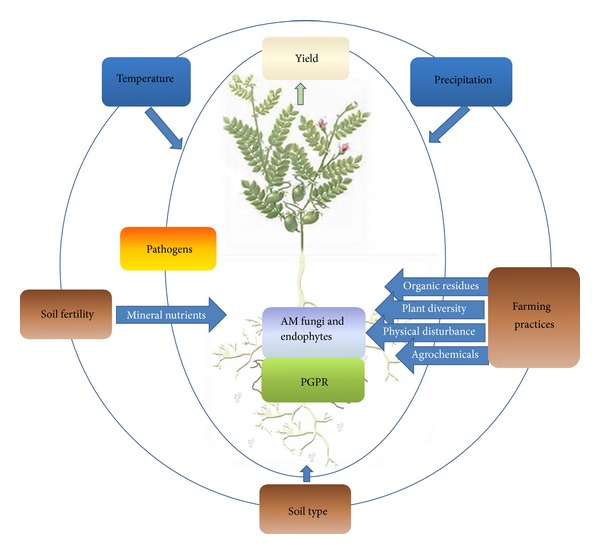
Graphical overview of the relationships between plant-associated microbial diversity, crop yield, and environmental conditions in agroecosystems as influenced by management.

In a world of seven billion people, the production of food and biofuel occupies an important proportion of the Earth's surface and therefore cropping systems must be efficient and sustainable. In light of the importance of soil microorganisms in the productivity of agroecosystems, the management of beneficial soil microbial diversity emerges as a new strategy for crop production in a changing world. This review considers the factors affecting the fungal resources relevant to agriculture and explores avenues toward the management of these resources to improve the efficiency of crop production. We propose a model where the plant is the key to the management of soil fungal resources and where the fungi living in close association with plant roots constitute the manageable resource (Figure 1).

In our view, arbuscular mycorrhizal (AM) fungi and fungal endophytes are the fungi that should be the target of management. We will review these key soil fungal groups, the plant mechanisms regulating them, and present different ways that could be used to improve soil health and, consequently, the efficiency of annual cropping systems. Although the concepts presented in this review are often relevant to all crops and production systems, they will be primarily illustrated with reference to dry land crops and cropping practices used in the cool and subtropical climates.

## 2. Important Soil Fungi in Agroecosystems

### 2.1. Arbuscular Mycorrhizal Fungi

AM fungi are ubiquitous in terrestrial ecosystems and form a symbiotic relationship with the roots of most plants [1]. They are obligate biotrophs requiring a plant partner for their carbon supply and are unable to complete their reproductive cycle without a host plant [2]. Initiation of the symbiosis can occur through the colonization of plant roots by germinating spores, hyphae, or infected root fragments [3]. Upon colonization, AM fungi form different functional structures in the root cortex of the host plant including arbuscules and hyphal coils (primary sites of nutrient exchange), vesicles (storage structures), and spores (reproduction) [1]. Through the AM symbiosis, the host plant is connected to extensive hyphal networks in the soil [4].

The primary function of the AM symbiosis involves a bidirectional transfer of carbon from the plant in exchange for soil-derived nutrients from the fungal partner [1]. Extensive networks of extraradical mycelium in the soil enable the fungus to uptake and rapidly translocate nutrients to intraradical arbuscules and hyphal coils and into the plant, thereby increasing the availability of soil nutrients in the soil to the host plant [1]. In addition, AM fungi can provide other functional benefits to the host plant such as improved water relations [5] and protection from pathogens and herbivores [6, 7]. The AM association is usually mutualistic, but evidence does suggest that it can range from parasitic to mutualistic [8].

AM fungi are also involved in several important ecosystem processes. They have a direct effect on plant productivity and have been shown to influence plant diversity and community structure [9–11]. In addition, the extensive mycelial networks produced by AM fungi coupled with the secretion of glomalin have a beneficial impact on soil health by improving the structural stability, quality, and water retention of soil [12, 13]. AM fungi also play an important role in the cycling of major elements such as carbon (C), phosphorus (P), and nitrogen (N) [14]. From an agroecological perspective, the functions and ecological services provided by AM fungi reveal the important impact these symbiotic organisms have on the productivity and sustainability of agricultural systems [15–17].

There are various abiotic and biotic factors that influence the distribution, growth, and function of AM fungi. These include abiotic factors such as soil chemistry (e.g., pH, nutrient availability, and pesticides [18, 19]), climatic variables (e.g., temperature, light, and precipitation [20–22]), and soil structure and stability [23, 24]. Biotic factors are primarily linked to the composition of the plant community as several studies have found that the diversity and assembly of AM fungal communities are strongly influenced by the plant community [22, 25–27]. Other biotic factors that have been shown to influence AM fungi are root predators [28], plant parasites [29], and herbivores [30]. Many of these abiotic and biotic factors are interrelated and interact synergistically to influence the habitat and in turn the composition and functioning of AM fungal communities.

In agricultural systems, many of these abiotic and biotic factors are modified by management techniques, which strongly impact AM fungal communities. Studies have shown that practices such as tillage and fallow [31, 32], monoculture cropping [33], and fertilization [34] all negatively influence the abundance and diversity of AM fungi. In general, agroecosystems have a lower AM fungal diversity compared to natural ecosystems [35] and this loss of diversity appears to be correlated with management intensity [36, 37].

### 2.2. Fungal Endophytes

Two important groups of non-AM fungi associated with plant roots are functionally defined as pathogens and endophytes. Both fungal endophytes and pathogens can colonize plant tissue, but, in contrast to endophytes, pathogens are able to cause disease in plants [38]. Pathogenicity is not exclusive to fungi, but in agricultural systems most plant diseases are caused by fungal pathogens [39]. Fungal pathogens have attracted much research attention because they are responsible for very important yield losses. Fungal pathogens are unwanted in agroecosystems and agronomic practices are aimed at controlling their abundance and their impacts.

Endophytic fungi are a group composed of very heterogeneous fungi that have been divided into two major groups: clavicipitaceous and nonclavicipitaceous endophytes [40]. Clavicipitaceous endophytes are a small group of fungi usually transmitted through seeds and that colonize the shoots of some grass species [40, 41]. The nonclavicipitaceous endophytes are a very diverse group of fungi (primarily ascomycetous) sharing the capacity to colonize the root systems of a wide range of plant lineages and which often have dark and septate hyphae [40]. While little is known about the ecology and functionality of endophytic fungi, a growing number of reports have revealed the beneficial services provided by endophytic fungi to host plants. The potential for commercial application of mutualistic endophytes with biocontrol abilities has promoted research in this field and several bioproducts for the control of plant diseases are already commercially available [42].

Many endophytic fungi have been reported to protect plants against diseases. For example, inoculation with* Beauveria bassiana* protected cotton and tomato against the pathogens* Rhizoctonia solani* and* Pythium myriotylum* [43].* Trichoderma atroviride* and* Epicoccum nigrum* also protected potato against* Rhizoctonia solani* [44].* Trichoderma* is a genus well known for having biocontrol activity against pathogenic species and some* Trichoderma* isolates are formulated and used as inoculants for the control of several plant diseases like onion white rot, Fusarium wilt of chickpea, and Fusarium crown and root rot of tomato [42, 44–48]. Different mechanisms are suggested to explain the protection of plants by their fungal endophytes [49] including competition for niche occupation and resource utilization [43], direct interaction [50, 51], or induced systemic resistance [43, 52].

Some fungal endophytes can also protect plants against abiotic stress created by drought [53], salinity [54], or toxic levels of metal [55], while others were reported to promote plant growth [52–54, 56]. The production of plant hormones and growth regulators appears to be an important mechanism by which fungal endophytes improve plant growth and yield under stressful conditions [54].

Accumulating evidence indicates a nutritional effect of soil fungal endophytes on their host plant (e.g., [57, 58]). Solubilisation of soil phosphorus appears to be involved in the improved plant P uptake mediated by fungal endophytes [54, 59]. In addition, enhanced mineralization is suggested to explain the role of fungal endophytes in plant nitrogen nutrition [49].

Understanding population dynamics and community structure of fungi in agricultural systems is necessary to minimize the damage from pathogens and optimize the benefits of mutualistic fungi. In addition to natural environmental fluctuations, anthropogenic activities can drastically affect fungal communities. Potent pathogens are carried across continents [60] and climate warming will shift the host range and fruiting date of some important fungi [61, 62]. In agroecosystems, cropping practices have profound and immediate impacts on the soil fungal community by modifying environmental factors such as soil pH, fertility, moisture, and plant cover. Among soil properties, pH is known as a major factor shaping the community of root-associated fungi [63, 64]. Soil nutrient availability and organic matter content are also thought to influence root endophyte diversity [65–67]. However, host preference is the most important factor in plant-fungal relationships [52, 63, 68] and crop selection likely has the strongest effect on fungal endophyte community composition in agroecosystems.

## 3. Mechanisms of Plant Control over Fungi

Plants coexist with a wide variety of beneficial and pathogenic fungi at all stages of their life. Plants actively interact with fungi using numerous mechanical and biochemical tools [131] and have evolved sophisticated strategies to shape the structure and function of their fungal environment [132]. Rhizodeposition is the process through which plant roots release organic and inorganic compounds that modify the physical, chemical, and biological properties of their soil environment [133–135]. Plant roots release a wide array of compounds that act as nutrient sources for soil fungi and as highly specific chemicals involved in diverse biological interactions [136, 137]. The secretion of carbon compounds derived from cortical and epidermal cells stimulates the proliferation of fungi outside, on the surface, and inside the roots [134]. An abundance of fungal growth on the root creates a barrier inhibiting the relative growth of pathogenic microorganisms through interspecific competition.

**Figure 2:**
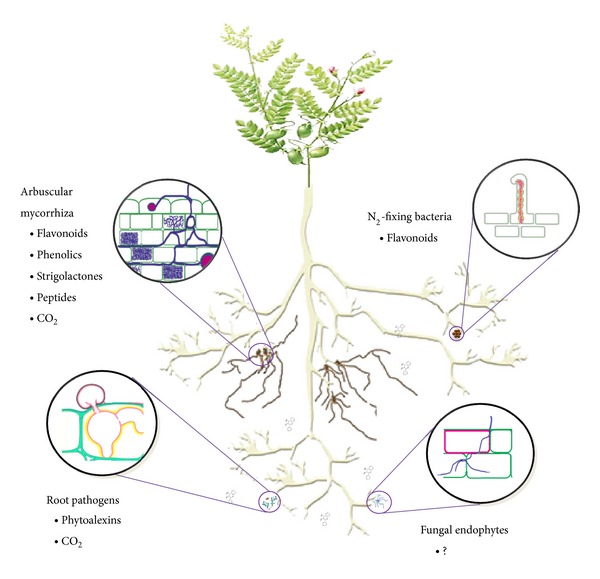
General overview of the bioactive phytochemicals involved in interactions between plants and soil microorganisms.

Several chemical pathways involved in the communication between plants and soil fungi have been identified and are illustrated in Figure 2. Phenolic compounds play key roles in presymbiotic stages of the AM symbiosis. They stimulate AM hyphal growth and branching [138]. Root symbioses are tightly controlled interactions. The extent to which root tissues are colonized by AM fungi and rhizobia is subjected to autoregulatory mechanisms preventing excessive colonization of the roots by the microsymbionts, thus preserving the symbiotic nature of the associations [139].

Plant hormones play a major role in the complex signalling and regulatory processes controlling plant-fungus interactions [140]. These include salicylic acid, ethylene, jasmonic acid, abscisic acid, gibberellic acid, auxin, cytokinin, strigolactones, and brassinosteroids. Salicylic acid is associated with the control of biotrophic plant pathogens while ethylene and jasmonates are involved in plant defence against necrotrophs [140]. Strigolactones are exuded into the rhizosphere under harsh environmental conditions and are known to stimulate hyphal branching of AM fungi and generally inhibit the growth of pathogenic fungi [141–143].

Plant proteins are also involved in interactions with soil fungi. Tryptophan dimers secreted from Bahia grass roots acted as a signal, stimulating the growth of AM fungal hyphae, under water-limiting conditions [144]. Peptides with hormonal activity are a component of the defence mechanism of plants [140]. Plant roots also secrete a wide spectrum of antimicrobial proteins such as chitinases that disrupt the cell wall and suppress the growth and function of pathogenic fungi [145, 146]. Extensin and other proteins identified in a root extract appeared to be involved in the suppression of AM fungal spore germination [147]. Furthermore, several types of volatile organic compounds (VOC) were found to trigger responses in insects but also to suppress the growth of pathogenic fungi, in particular* Fusarium* spp. [148, 149]. Plant-fungus interactions are highly complex and involve hormonal, mechanical, and biochemical factors.

Plants are more than a mere source of nutrients for soil fungi. They have coevolved with specific fungi and specific soil fungal communities, which led to the emergence of various lifestyles and forms of coexistence in the plant kingdom. For example, plants from the Fabaceae, such as pea, bean, and lentil, are associated with AM fungi [150]. Wheat, barley, rye, and oat are members of the Poaceae and they associate with AM fungi [151, 152], but as members of the subfamily Pooideae, they rarely respond to the symbiosis [153]. The Brassicaceae, including oil seed canola or mustard, do not associate with AM fungi or rhizobia [154].

Plants influence soil fungal diversity. The cultivation of mycorrhizal crops increases the inoculum density, which promotes the formation of mycorrhizal symbioses in the following seasons. Research has revealed that when a mycorrhizal crop is cultivated in rotation after a nonmycorrhizal crop, root colonization and symbiotic contributions to plant growth are delayed as a result of decreased levels of inoculum in the soil [111]. The genotypes and species of these broad taxonomic groups of plants have different phytochemistry [147, 149] and influence the soil microbial communities in slightly different ways [155].

## 4. Management of Soil Fungal Resources

### 4.1. Management through Genetic Selection of Plants

Technologies for agriculture have emerged from research on the biochemistry of plant-microbe regulation. The use of formulations of flavonoids or lipochitooligosaccharides at seeding now enhances crop production in fields of the Canadian prairies and elsewhere through the use of products such as PulseSignal II or Optimize (Novozymes BioAg Group). The mechanisms plants implement to manage their microbial environment are complex [131, 132] and as difficult to manipulate as they are finely regulated. The intraspecific variation observed in the profile of plant signaling phytochemicals [147, 149] and concurrent fungal environment [155] suggests the possibility of selecting crop plants with special compatibility with beneficial fungi. The selection of plant genotypes resistant to pathogens has already led to important progress in phytoprotection [156] and points toward plant management as a key to managing soil fungal resources in agroecosystems. Selection of plant genotypes that have favourable compatibility with beneficial soil fungi is possible, as shown by variation in the compatibility of certain genotypes with beneficial fungi that were found in the studies listed in Table 1.

Growing crop varieties with improved compatibility with beneficial soil fungi can be a powerful way to manage soil fungi and a good strategy to enhance soil nutrient use efficiency in agroecosystems. Some studies suggest that modern breeding programs conducted in highly fertilized systems may have produced cultivars with a high level of dependence on fertilizer and a diminished capacity to form symbiotic relationships with beneficial soil fungi [69–71, 100, 157]. However, this hypothesis was disproved by a meta-analysis evaluating the importance of the year of release on mycorrhizal responsiveness, AM fungal root colonization, and P efficiency [158]. There is little evidence to support a negative impact of plant breeding on AM formation and function. In fact, the prolific growth of AM fungi that can be seen in the rhizosphere of certain recent cultivars [72] could suggest that modern plant breeding approaches have improved the microbial associations with crop roots.

Plant genotypes differentially influence the soil microbial communities of agricultural fields [155]. Mixtures of cultivars have led to yield stability over a range of environmental conditions and sustained higher productivity than monocultures [159]. These effects were attributed to crops maintaining health-promoting soil microbial communities [109]. Mixtures of cultivars create diversified niches that maintain a higher diversity of beneficial soil microorganisms with host preference [160] and functional complementarity [124].

Overall, breeding crop varieties with an improved ability to interact with beneficial soil fungi appear to be a logical approach to enhance crop yield. Targeting plant genes responsible for beneficial interactions with soil fungi should improve the nutrient efficiency of crops and reduce the environmental impacts of fertilization, as well as farm input costs, leading to more sustainable production systems.

### 4.2. Management through Rotation

Certain agronomic practices are designed to manage biodiversity in the agroecosystem by enhancing diversity and repressing pests and disease outbreaks (Table 2). Among these practices, rotating crops is one of the more traditional and effective ways to diversify the microbial community, reduce the impact of diseases and weeds [101], and thus increase yields. The value of a cropping system depends on a number of factors including the genotype and crops included in the rotation [102], the sequence and frequency of the crops [103], the length of the rotation [161], the management history [162], and soil characteristics [163]. Overall, these factors impact the soil microbial community in different ways.

Intercropping systems and crop rotations offer opportunities for a better management of soil fungi. Using mixtures of different cereal genotypes [104, 109] or crops such as wheat, barley, canola [105], clover, and alfalfa [106] can enhance productivity by reducing weeds and disease incidence at the system level. Also, changes in the frequencies of cultivars [103, 104] over time can influence the incidence of stem and root rot diseases in the rotation system and enhance yield stability. For example, corn grain yield can increase linearly in relation to the number of crops included in the rotation up to twice the yield of the monocrop when three rotation crops and three cover crops are included in the cropping system [107]. Certain crops in the rotation are better than others and it can be complicated to determine what the optimal rotation sequence to maximize benefits is [103]. Soil factors are also important to consider in the design of rotation sequences (e.g., soil-water stable aggregation, soil organic C, and the carbohydrate composition of the surface layer) as these parameters also affect the abundance, diversity, and distribution of the fungal community [108]. In most cases, monoculture negatively affects microbial biomass and diversity [164, 165]. Diversifying the crops used in rotation increases the taxonomic and functional diversity of soil fungal communities [166]. In addition, microbial activity and substrate utilization are significantly affected by crop rotation [110]. Different crops provide different organic residues, which can result in a diverse food base that promotes fungal diversity and activity and increases soil fungal biomass and N mineralization [167]. Interestingly, the biochemical composition of some plant tissues can modulate the fungal associations. Plants of the Poaceae are particularly rich in pentoses, which are the main energy source of soil fungi. So it is not surprising that many fungi are associated with cereals.

Diversifying crop rotations also decreases disease pressure in agroecosystems by disrupting the life cycle of pathogens associated with a particular crop or plant genotype. The length and level of crop diversity are key factors for the success of a cropping system. Short rotations are more susceptible to diseases and produce lower yield than longer rotations [161]. Other factors to consider in the design of crop rotation systems include the ability of plant pathogens to use alternative host plants or remain dormant in the soil for long periods [168] and allelopathy and autotoxicity of crops [161]. Selecting plants that are not alternate hosts for pathogenic fungi in other components of the rotation is important to reduce yield losses due to diseases. However, some pathogens can persist in the soil for several years as spores or other dormant structures, in absence of a host plant [168]. In addition, monocultures negatively affect fungal biodiversity by selecting for virulent pathogens, which then have a competitive edge and increase disease severity. In a continuous-pea rotation grown in the Canadian prairie, severe Fusarium root rot injury was related to a reduced soil microbial community and lower abundance of beneficial Gram positive bacteria and AM fungi [165]. In some cases, continuous cropping has increased the abundance of antagonistic microorganisms and reduced pathogen populations, mitigating the impact of take-all in wheat [102], but as a general rule, at least three and possibly more crops should be included in cropping systems [161]. The inclusion of cover crops in cropping systems is particularly effective in reducing disease incidence [110].

In semiarid cold and subtropical steppes, farmers have traditionally grown cereals in alternation with summer fallow. This consists of keeping the soil bare using tillage or herbicides during a growing season. In the last two decades, broadleaf crops such as field pea, lentil, chickpea, canola, and mustard were introduced in wheat-based rotations in the semiarid area of the Canadian prairie to replace summer fallow, which lost relevance with the development of no-till systems for soil moisture conservation [107, 169]. Crop diversification with broadleaf crops, especially pulses, has the benefit of increasing grain yield and protein content of the wheat crops following in rotation, partially due to residual soil N from biological fixation [103].

Canola and mustard are nonmycorrhizal plants that do not associate with rhizobacteria. These crops also require the use of more N and S fertilizers; however, the productivity and value of these crops compensate for the larger investment in fertilizers. Despite the economic benefit of these crops, having nonmycorrhizal plants in the crop rotation may reduce AM fungal populations and delay mycorrhizal formation in the following crop [170, 171], which may impact AM dependent crop plants. Clearly, there are many factors to consider in the design of ecologically sustainable and economically viable crop rotation systems.

### 4.3. Management through Biochemical Amendments

The use of biologically active chemicals is an alternative approach to managing the structure and function of soil fungal communities. Plants naturally release a wide spectrum of bioactive phytochemicals that modify their microbial environment. The phytochemicals contained in plants vary with the species, genotype, tissue, physiological stage, and environmental conditions [147, 149, 172, 173]. The application of plant tissues containing certain phytochemicals as dried organic amendment or green manure can effectively reduce the inoculum of soil borne plant pathogens and stimulate the growth of beneficial fungi. For example, incorporating the tissues of certain legumes into infected soils has shown the potential to control parasitic nematodes and reduce gall number in tomato [174]. These legumes contain bioactive phytochemicals that negatively impact plant-parasitic nematodes. Plants of the Brassicaceae contain glucosinolates and have long been known for their activity against fungal pathogens.* Brassica napus* seed meal applied to orchard soil reduced the infection by fungal pathogens (*Rhizoctonia *spp.) and parasitic nematodes (*Pratylenchus *spp.) of apple roots [175]. The control of* Rhizoctonia* root rot of apple by* B. napus* was attributed to the modification of the bacterial community structure and the induction of plant systemic resistance [175]. This suggests that stimulating soil fungal communities by the addition of bioactive amendments may be an effective way to manage soil fungal communities and control pathogens [176].

The production of bioactive VOC by plants can trigger responses in the organisms surrounding them and inhibit certain pathogens. Changes in the profile of VOC by plants are generally a response to pathogenic invasion. For example, the profile of VOC from chickpea was correlated with Ascochyta blight severity [149]. The VOC of chickpea, in particular trans-2-hexenal and 1-hexanol, were much more potent against the causing agents of Fusarium head blight than wheat VOC [149]. This provided an explanation for the susceptibility of wheat and the resistance of chickpea to these pathogens [149]. Selection of genotypes based on VOC production may be a strategy to increase disease resistance in crop rotations.

### 4.4. Management through Inoculation and Soil Management

Rhizobial inoculants have been used in agricultural systems for decades and are proven efficient tools to manage beneficial soil microbial diversity. Inoculation of crops with selected plant growth promoting microbial strains (e.g., PGP rhizobacteria and AM fungi) is a strategy that can easily be integrated into cropping systems [177, 178]. Although simple, inoculation of crops can be unreliable. Competition among microorganisms in the soil system can be intense and introduced organisms may not live long enough to produce the desired effect, especially if their niche is not unique. The combination of inoculation along with certain agronomic practices may increase the probability of beneficial effects from inoculants. Practices that modify the soil environment in a way that benefits the introduced microorganisms may increase the value of inoculants.

Soil properties can modify the influence of fungi on plants and management practices that modify soil properties could be used to maximize the beneficial effects of inoculants. Because soil organic matter (SOM) controls many soil properties [179], the management of SOM appears to be a key to managing soil microorganisms. Amending the soil with organic materials and adopting conservation tillage practices are strategies that most effectively influence SOM.

Fresh organic matter and manure have a stronger effect on microbially mediated soil structuration than stable organic matter, but the effect of the latter is long-lasting particularly if large amounts are applied. Organic amendments contain energy and nutrients favouring fungal proliferation and are also rich in functional groups that can adsorb nutrients and retain water. This increases the soil nutrient pool and soil moisture levels, which further supports the growth and function of plants and fungi.

The addition of fresh organic material can immediately boost the performance of inoculants in annual cropping systems. For example, the addition of manure to soil improved the contribution of fungal biocontrol agents to plant health [122] and the plant-growth-stimulating effects of AM fungi [123]. Organic amendments benefit the microorganisms using them by providing a source of nutrients and energy, but the positive effect of organic amendments has also been attributed to their impact on soil physical quality [122]. The stimulation of fungal growth by organic amendments triggers the production of aggregate-stabilizing fungal filaments and exopolysaccharides that structure the soil matrix, which increases its porosity and has a positive effect on gas exchange and water infiltration and retention.

In regions where sources of organic amendments are not readily available such as the intensive grain producing steppes, no-tillage practices are effective methods for soil moisture conservation and increasing SOM levels [180]. Soil tillage has tremendous effects on soil physical and biological properties by homogenizing the soil matrix and stimulating mineralization, which in the long term reduces the level of SOM [180]. Consequently, the influence of no-till practices on soil physical properties is in many ways similar to the influence of organic amendments. Soil aggregates are conserved in the absence of tillage and the soil is well structured and porous. The organic matter is preserved in stable aggregates favouring SOM accumulation, which further improves soil porosity, aeration, and water infiltration and retention. The heterogeneity of the soil in the absence of tillage leads to the development of a variety of niches allowing the establishment of highly diverse microbial communities. However, the effect of no-till on SOM accretion is slow and develops through decades after the abandonment of intensive tillage practices [180]. The addition of organic amendments to soils with suboptimal physical properties is useful in accelerating the establishment of soil physical conditions hospitable to beneficial microorganisms.

While the combined use of organic amendments and inoculants can increase the performance of PGP microorganisms in cultivated fields, excessive rates of organic amendments may also be inhibitory to certain PGP microorganisms. For example, high concentrations of compost can inhibit AM fungi, whereas low rates are beneficial [124]. In addition, amendments used to create conditions favourable to crop plants may negatively impact the beneficial microbial associates of plants that are adapted to soil conditions suboptimal for production. This was shown to be the case for certain AM fungi, which had a reduced ability to colonize their host after a saline-alkali soil was amended with gypsum [181]. Although the soil conditions conducive to biological activity and biodiversity may be suboptimal for certain microorganisms with PGP activity, the maintenance of soil physical quality should favour the survival and functional activities of most PGP microorganisms introduced in agroecosystems through inoculation.

## 5. Influence of Agrochemicals on Soil Microorganisms

Managing the soil environment through the use of agrochemicals is often secondary to the primary goal of these products, but they are widely used and can strongly influence soil microbial communities. On the Canadian prairie, 73% of the land in crop production receives chemical inputs in the form of pesticides and/or inorganic fertilizers [182]. Most production requires fertilizer with inputs of 1.3 million metric tonnes of N and 0.48 million metric tonnes of P applied annually [183]. With this level of inputs going onto the soil it is important to understand and manage the effects these chemicals have on the soil environment.

There are many different fertilizer formulations available and some include amendments that directly affect and inhibit microbial activity [126]. Soil pH can be affected by different factors including the use of inorganic N fertilizers. The majority of the N applied is in the form of granular urea or anhydrous ammonia, both of which have been found to be less acidifying to soil than ammonium sulphate or ammonium phosphate formulations. The level of acidification resulting from ammonium fertilizer varies with soil characteristics and cropping systems [184], but there is considerable evidence of soil acidification due to N fertilizer use in the Canadian prairies despite the high buffering capacity of these soils [127, 185, 186]. The drop in pH can be alleviated by liming the soil, but the associated costs limit the use of this practice [187]. In general, long term N use lowers soil pH and in turn has a negative impact on certain soil microbial groups, especially actinomycetes and denitrifying bacteria. In general, fungi can tolerate a wider range of pH than bacteria [188]. Lower pH does not appear detrimental to fungi and may sometimes increase their abundance [127].

With the exception of soil fumigants and certain fungicides, pesticides appear to have a limited effect on soil fungi [189]. Recent studies have demonstrated that pesticides have a minimal effect on soil fungi when they are applied at the recommended doses [190]. However, pesticides may influence the function and ecological processes associated with the soil fungal community. For example, there is some evidence that pesticides can effect soil biochemical reactions, especially related to nutrient cycling [118, 191]. In addition, the application of fungicides against foliar disease influences not only the production of VOC in the aboveground tissues, but also the production of these antimicrobial phytochemicals in the roots [149]. As a result, foliar applied fungicides can significantly affect plant-pathogen interactions in the rhizosphere. The widely used herbicide glyphosate can also modify the structure of rhizosphere fungi under certain cropping practices [119].

Since fertilizers and pesticides are commonly used together in conventional cropping systems, it is important to understand the interactive effects of these agrochemicals. A study in the Canadian prairies found that, in the short term, fertilizers and herbicides have beneficial or minimal effects on soil microbiological characteristics [120]. However, over time some deleterious effects on soil microorganisms and their associated biological processes were observed indicating the cumulative effect of repeated applications of agrochemicals [120]. Meanwhile, other studies have reported interactive effects of pesticides and soil fertility on soil microbial communities. For example, herbicides had a more pronounced effect on soil microbial community structure in soils with low fertility [192] and in crops not fertilized with N [119]. Furthermore, fertilization can influence the degradation of pesticides and modify their nontarget effects on soil microbial communities [121].

The influence of agrochemicals on important soil fungi is complex and difficult to predict, further increasing the difficulty involved in the management of soil fungal resources. Agrochemicals are abundantly used in annual crop production systems and are considered a necessity to achieve desired crop yields. Future research should focus on optimizing pesticide and fertilizer applications that promote beneficial soil fungi and their associated biological processes to encourage more sustainable agroecosystems that are less dependent on conventional agrochemicals.

## 6. Conclusion

The soil fungi that have the strongest influence on plants reside in the rhizosphere and it appears that plants can be used to manipulate these fungi in order to improve soil health and the efficiency of annual cropping systems. In this context, the traditional practice of crop rotation can be used as a basic strategy to increase diversity in the rhizosphere and prevent the build-up of pathogens. Future approaches to complement crop rotations will likely include the use of cultivars with specific compatibilities with beneficial fungi. In addition, biotechnologies based on the use of bioactive phytochemicals and fungal inoculants are currently available and are being diversified and refined. Combining inoculation with practices that create conditions favourable to the survival and activity of the desirable fungi will be an effective strategy to increase the value of inoculants. Despite the complexity of the soil ecosystem, it is possible to manage soil fungal diversity in order to promote more sustainable and productive agroecosystems. As global change dictates the need for more efficient cropping systems, the management of beneficial fungi offers many opportunities.

## Figures and Tables

**Table 1:** Reports of intraspecific genetic variation in the ability of crop plants to host beneficial fungal endophytes, a necessary condition for genotype selection in genetic improvement programs.

Microorganism	Type and function	Host plant	References
AM fungi	Symbiotic soil fungi improving the ability of host plants to extract soil nutrients	Wheat (*Triticum* spp.)	[69–82]
		Barley (*Hordeum vulgare* L.)	[83]
		Triticale (×*Triticosecale*)	[82]
		Oats (*Avena* spp.)	[84]
		Maize (*Zea mays* L.)	[85–90]
		Rice (*Oryza sativa* L.)	[91, 92]
		Soybean (*Glycine max* (L.) Merr.)	[88, 93]
		Onion (*Allium* spp.)	[94, 95]
		Tomato (*Lycopersicon esculentum* Mill.)	[96]
		Peanut (*Arachis hypogaea *L.)	[97]
		Marigold (*Tagetes *spp.)	[98]
		Pepper (*Capsicum annuum* L.)	[99]

*Acremonium *	Fungal shoot endophyte increasing plant vigor, resistance to insects, and modifying water relations	Wheat (*Triticum* spp.)	[100]

*Neotyphodium *	Fungal shoot endophyte improving plant tolerance to stress	Wheat (*Triticum* spp.)	[100]

**Table 2:** General effects of agronomic practices on soil fungal diversity and abundance, disease incidence, soil fertility, crop nutrient use efficiency, and crop growth and yield.

Source of effects	Biodiversity level	Crop growth and productivity	Disease, pests and pathogens	Microbial abundance	Soil fertility	Nutrient use efficiency	References
Biodiversity management							
Crop rotation	+^a^	+	−	+			[101–108]
Cultivar mix	+	+	−	+			[103, 104, 109]
Intercropping	+	±	−	+			[106]
Cover cropping	+	±	−	+			[106, 107, 110]
Nonmycorrhizal crops	−		+			−	[111]
Transgenic crops	0	±	−	0			[112–117]
Pesticide use	0	+	−	0		−	[118–121]
Weed control	−	+		−		+	[118, 120]
Inoculants		±	+		+	+	[80, 91, 97, 98, 122, 123]
Soil management							
Organic amendments	+	+		+	+	±	[102, 124, 125]
Nitrogen fertilizers	±	+		+	+	−	[126, 127]
Mineral fertilization		+		±	+	−	[120, 126]
Tillage	±	±	±	±	±	±	[31, 128–130]

^a^+ (positive to no effects), 0 (negligible effects), − (negative to no effects), and ± (variable effect).
